# Cell Docking, Movement and Cell-Cell Interactions of Heterogeneous Cell Suspensions in a Cell Manipulation Microdevice

**DOI:** 10.3390/s111009613

**Published:** 2011-10-12

**Authors:** Fei-Lung Lai, Yu-Hung Wang, Yu-Wei Chung, Shiaw-Min Hwang, Long-Sun Huang

**Affiliations:** 1 Institute of Applied Mechanics, National Taiwan University, 1 Sec. 4 Roosevelt Road, Taipei 10617, Taiwan; E-Mails: f95543034@ntu.edu.tw (F.-L.L.); yhwangx@mems.iam.ntu.edu.tw (Y.-H.W.); ywchung@mems.iam.ntu.edu.tw (Y.-W.C.); 2 Bioresources Collection and Research Center, Food Industry Research and Development Institute, 331 Shih-Pin Road, Hsinchu 300, Taiwan; E-Mail: hsm@firdi.org.tw

**Keywords:** Natural Killer cells, microfluidics, MEMS, cell-cell interaction, cytotoxicity, microchannel

## Abstract

This study demonstrates a novel cell manipulation microdevice for cell docking, culturing, cell-cell contact and interaction by microfluidic manipulation of heterogeneous cell suspensions. Heterogeneous cell suspensions include disparate blood cells of natural killer cells and leukemia cancer cells for immune cell transplantation therapy. However, NK cell alloreactivity from different healthy donors present various recovery response levels. Little is still known about the interactions and cytotoxicity effects between donor NK cells and recipient cancer cells. The cell-based micro device first showed the capability of cell docking, movement, contact and cell-cell interaction with respect to cell cytotoxicity of NK cells against cancer cells. With various flow tests for live cell loading, flow rates of 10 μL/h were chosen for injection in the central and side flows such that both types of suspension cells could be gently docked at the gap structure in a reaction zone. The trapping number of particles and cells was linearly proportional to the gap length. Finally, the cytotoxicity of around 40% was found to be similar in the case of dilute cells and a large cell population. As a result, the cell manipulation microdevice has been validated for live suspensions of natural killer and cancer cells, and exhibited the capability to measure the cytotoxicity of dilute cell suspensions.

## 1. Introduction

There is growing awareness that natural killer cell transplantation therapy has the potential to be an effective treatment for some cancer diseases. A growing volume of evidence from clinical trials indicates that this therapeutic approach has promise in the treatment of leukemia [1–3]. The common clinical treatments of leukemia include radiation and chemotherapy, stem cell transplantation, and immune cell transplantation. Although new chemotherapeutic agents are still used in therapy, a number of patients suffer serious side effects after treatment. From one third up to one half of children with acute myeloid leukemia suffer relapses [4,5]. Hematopoietic stem cell transplantation (HSCT) is another choice for many hematologic diseases, but requires a strict match of human leukocyte antigen (HLA). There is only a 25% chance that one will inherit all the same HLA haplotypes from the parents. Therefore, this approach restricts donor selection, resulting in its limited applications. Moreover, after the treatment of hematopoietic stem cell transplantation, relapse may also lead to high mortality of up to 16% [2]. Although natural killer (NK) cell transplantation is still a developing therapy approach, it is expected to decrease the risk of relapse after treatment.

Unlike the cytotoxic T lymphocytes of the adaptive immune system, NK cells are part of the innate immune system and play an important role in the first line of defense against diseases. NK cells exhibit spontaneous cytotoxicity against pathogen-infected or malignant cells. Having no antigen-specific recognition, they require direct contact with target cells. The major mechanism of cell killing is executed mainly through granule exocytosis pathway where the perforin and granzyme B contents of the granules from NK cells are released into the immunological synapse [6]. NK cells provide an immediate natural response and rapid cytolytic action for a wide range of malignancies. As a result, NK cells offer promise as good candidates for transplanted cells from healthy donors. Moreover, NK cells do not require a HLA match, unlike the transplantation of hematopoietic stem cells. Therefore, the restricted selection of donors for cell transplantation is relaxed, and a potential for widespread is highly expected.

However, NK cell alloreactivity from different healthy donors presents various levels of recovery response [7–9]. As yet there is no appropriate assessment for matching various healthy donors with recipients prior to NK cell transplantation. As a result, no devices or schemes are used to allow cell-cell interaction to gain an insight of cytotoxicity of NK cells against cancer cells.

Microfluidic systems provide powerful tools and have attracted great attention for cell analysis. Microfluidic technology has been used in recent years to analyze the biophysical and biochemical properties of cells. Studies report that microfluidics in micro-cell devices can provide cell transportation [10–13], immobilization [14–16], trapping [17–19], manipulation [20,21], and cell interaction and communication [22,23] for biophysical and biochemical analysis of cells.

This study proposes a cell-based microdevice that provides a platform for cell assessment of the interaction between effector NK cells and target cancer cells. The cell-based microdevice allows suspension NK and cancer cells to be conjugated, cultured, and to interact biologically for analysis and assessment. Cell-cell interactions can also be monitored in real-time for investigation of cell activity and cytotoxicity.

## 2. Experimental

### 2.1. Sample Preparation

Heterogeneous blood cells include suspension natural killer and leukemia cancer cells. The natural killer and cancer cells were effector cell cell-line NK92 and target cell cell-line K562, respectively. Both cell types, provided by the Bioresource Collection and Research Center in Taiwan were cultured *in vitro* in an environment with 37 °C, 5% CO_2_, and 95% of relative humidity. The culture media for K562 cells is Iscove’s modified Dulbecco’s medium (IMDM, Invitrogen) supplemented with 10% fetal bovin serum (FBS, HyClone), 3.02 g/L (grams per liter) sodium bicarbonate (Sigma), and 1% penicillin-streptomycin (HyClone). The NK92 medium consists of α-modified minimum essential medium (α-MEM, Invitrogen), 1.5 g/L sodium bicarbonate (Sigma), horse serum 12.5% (HS, Invitrogen), 12.5% FBS, 0.2 mM inositol (Sigma), 0.1 mM 2-mercaptoethanol (2-ME, Sigma), 0.02 mM folic acid (Sigma) and 100 U/mL recombinant IL-2 (PeproTech, and 1% penicillin-streptomycin (HyClone).

The NK92 and K562 cell sizes in this study were found to be around 10 μm and 20 μm in diameter, respectively. To validate the device for cell transportation, trapping and microfluidic manipulation, micro particles were used to simulate the cell behaviors in a reaction zone. Micro particles made of polystylene and with diameters of 10 μm and 20 μm were selected, and the concentration tested was around 1 × 10^5^ particles per mL.

For cell identification, cancer cells of K562 were chosen to be labeled with quantum dots (QDs) using Qtracker 525 (Qtracker® 525 cell labeling kit, Invitrogen). The 10 nM labeling solution was prepared according to the manufacturer’s instructions. Harvested K562 cells (1 × 10^5^) were added to the labeling solution (0.2 mL). After incubation at 37 °C for 60 min, most QDs were delivered into the cytoplasm of live cells. By washing out free QDs with phosphate buffered saline (PBS), the cancer cells with QD inside were retained in a culture plate. Finally, the QD-labeled K562cancer cells were excited by laser at a wavelength of 406 nm for cell identification and visualization. The cells labeled with QDs were identified by the green fluorescence exhibited at a peak wavelength of 525 nm.

### 2.2. Concept and Design

**Figure 1.**
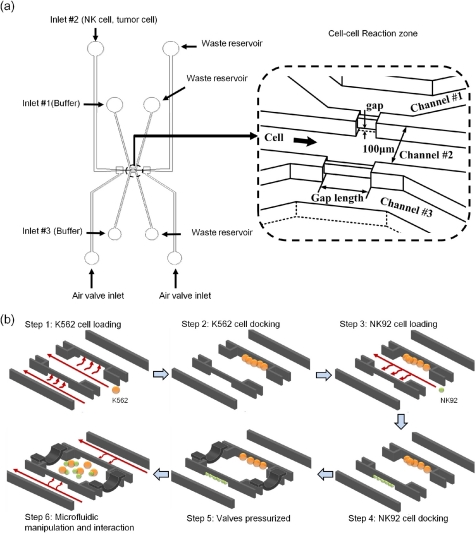
**(a)** Schematic representation of the microfluidic cell reaction chip. It includes micro-channel layer and air valve layer combined together. The reaction zone in the micro-channel layer is used to trap cells and observe cell-cell interaction. **(b)** The operation procedure of cell docking, movement, and cell-cell contact by microfluidic manipulation is demonstrated. K562 and NK 92 cells are sequentially docked at the right and left sides of a reaction chamber, respectively. In step 6, the membrane is pressed down by air pressure to form a micro enclosure and then cell movement toward the central area is made for cell-cell contact by microfluidics at left and right channels.

Figure 1(a) illustrates the design of the cell manipulation microdevice. The device is designed to have three inlets and three microchannels of which the central one allows for cell loading and the other two are injected with the medium flow in microchannels. Both suspension blood cells of natural killer and cancer cells are individually injected and transported into a reaction zone which allows for cell docking, movement, and cell-cell interactions. Figure 1(b) shows the device operation for cell docking, movement, and cell-cell contact by microfluidic manipulation. Both cancer and natural killer cells are sequentially docked at the right and left sides of a reaction chamber. Firstly, as shown in step 1 of Figure 1(b), the K562cancer cells were loaded into the central microchannel by pumping microfluidics in the central and left channels. Because of the fluidic pressure difference, the cancer cells were pushed to the right side and docked at the gap in a reaction zone. Likewise, natural killer cells were then loaded into the central channel at a fixed flow rate in step 4, while the flows of both side channels are maintained stationary. A pressure difference occurred between channels to allow NK92 cell docking at the left in a reaction zone. In step 5, the PDMS-based air valves located near the reaction zone along the central channel was pressed down by air pressure to form a micro enclosure. Finally, cell movement toward the central area was allowed for cell-cell contact by microfluidic shear forces at the left and right channels in step 6.

The reaction zone is designed with 30 μm in height and 100 μm in width. Meanwhile, there are small gaps of 1 μm in height on both sides in a reaction zone connecting to the left and right channels. The gaps allow both cells to be trapped at both sides of the reaction zone by the pressure difference between channels. Cell numbers and cell ratio were examined to quantitatively determine the cytotoxicity in this study. Meanwhile, cell sizes and lengths of the gaps may determine the number of trapped cells. As a result, the lengths of the opening gap were designed with 100, 200, 300, and 400 μm in a reaction zone.

In such an approach cell manipulation, cell deformation, cell characteristics, and cell activity are achievable in this cell manipulation microdevice. As docked inside the chamber, cells exposed to a microfluidic shear force tend to be deformed or squeezed under the gaps to pass into neighboring channels. The microfluidics-induced force is considered in laminar flow. First of all, the pressure difference across docked cells can be described from the Bernoulli equation [24]:

(1) $$\frac{\Delta P}{\rho }=\left(\frac{{\bar{V}}_{c}^{2}}{2}-\frac{{\bar{V}}_{g}^{2}}{2}\right)+h_{l_{T}}$$

where ΔP is the pressure difference between channels, *ρ* is the density of the medium, *V̄_c_* is the average velocity at the cross section of the channel, *V̄_g_* is the average velocity at the cross section of the gap and *h*_*l*_T__ is the total head loss. The total head loss can be described as:

(2) $$h_{l_{T}}=h_{l}+\sum h_{l_{m}}=f\frac{L}{D}\frac{{\bar{V}}_{c}^{2}}{2}+K_{c}\frac{{\bar{V}}_{g}^{2}}{2}$$

where *h_l_* is the major loss, *h*_*l*_*m*__ is the minor loss, *f* is the friction factor, *L* is the channel length, *D* is the hydraulic diameter of rectangular duct and *K_c_* is the contraction loss coefficient. The dynamic pressure terms, major loss and minor loss can be calculated from the parameters in Equations (1) and (2), respectively, where the major loss has much more portion of influence than the minor loss and dynamic pressure terms. (See Table S1 in the Supplementary Information). Meanwhile, the parameter *f* and parameter *V̄_c_* are related to Reynolds number and flow rate, respectively, and Equation (1) can be simplified to:

(3) $$\Delta P=32\frac{\rho \gamma L}{D^{2}A_{c}}Q_{\mathrm{gap}}$$

where *γ* is the kinematic viscosity, *A_c_* is the sectional area of the channel, and *Q_gap_* is the flow rate across the gap. The pressure difference is directly related to the flow rate, and both cells are docked due to the pressure force. As the flow rate is increased, high pressure difference leads to cell deformation and cell-squeezed passage.

A shear force *F* acting on the cells docked beside the gap is given by the Stokes equation [25]:

(4) $$F=6\pi \mu r{\bar{V}}_{g}=6\pi \mu r\frac{Q_{\mathrm{gap}}}{A_{g}}$$

where *μ* is the fluid viscosity, *r* is the radius of the cell and *A_g_* is the sectional area of the gap. From Equations (3) and (4), the flow rate *Q_gap_* is the dominant parameter in this experiment. An appropriate flow rate allows cell docking, and maintains cell activity.

### 2.3. Device Fabrication

**Figure 2.**
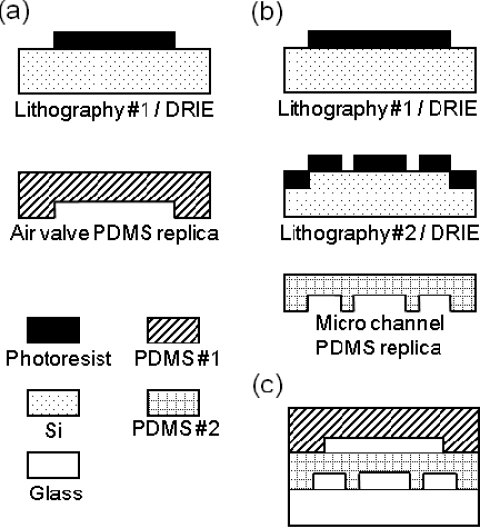
Schematic illustration of the fabrication process of the micro chip and the completed device. The manufacturing processes of **(a)** air valve replica and **(b)** micro channel replica.

The cell device starts with a glass substrate bonded with two polydimethylsioxane (PDMS)-based layers. The first layer of PDMS-based microchannels was made for cell loading and cultured medium flows, and bonded onto the glass substrate. The top layer of structured PDMS is used as air valves to be pneumatically pressurized and deformed, thus nearly forming a micro environment in a reaction zone. The elastic PDMS-based layers were replicas made from micromachined silicon casts. Figure 2(a) and (b) illustrate the manufacturing processes. Two silicon substrates were chosen to be molds for microchannels and air valves. Firstly, thick photoresists (S4620) were coated to be masks on silicon substrates for subsequent a deep reactive ion etch (DRIE) process. The depths of the silicon etch were controlled to be around 30 μm for microchannel, and 1 μm of another etch for the gap structures. Likewise, an additional silicon substrate etch was also made for the air valve mold structure. The PDMS mixture was poured onto the microstructure Si molds, and then cured for hardening. After the oxygen plasma surface treatment of the glass substrate and PDMS surfaces, the bonding between the glass substrates and PDMS layers was easily applied. Finally, the cell-based device was successfully made and formed with dimensions of 5.3 cm in length, 1.8 cm in width, and 0.3 cm in height.

### 2.4. System Setup

The entire system includes a cell device, an incubation chamber, a medium and drug supplying fluidic system, and a real-time monitoring system. Microparticles were first tested to simulate docking of live cells. Microparticles and live cell samples were loaded by the syringe pump with a 1% PBS solution at a rate of 10 μL/h. All of the experimental procedures were recorded with the real-time monitoring system, and incubated in a chamber which maintains the system at an appropriate temperature of 37 °C and a gas mixture of 5% CO_2_. Meanwhile, the real-time monitoring system was used with an inverted microscope (Olympus, IX-71) equipped with a digital camera (Olympus U-CMAD3). Additionally, the air valves of the cell device were connected to an air pump for membrane deformation. The wavelength that excites PhiPhiLux-G2D2 dye for identification of cell apoptosis is 552 nm.

### 2.5. Flow Cytometry Analysis Assay

A conventional flow cytometry analysis assay that requires a large amount of cultured cells was performed in this study for comparison with the cytotoxicity of the new cell-based device. Cancer cells (K562) labeled with QD 525 were co-cultured with live NK cells (NK92) in a conventional culture dish. With such a mixture and co-culture, the effector NK cells perform cytolytic action on the target cancer cells (K562). The co-culture ratio of effector NK 92 to target K562 was around 2 at 37 °C in air for 2 hours [2,26]. To discriminate the spontaneous cell death of target K562 cells from the cytotoxicity of natural killer cells, the target K562 cells were incubated alone in a medium for NK culture. With this experiment, the spontaneous cell death of target cancer cells can be excluded from the observed total cell death. Later, the solution of supernatant was removed and replaced with the indicated caspase substrate PhiPhiLux-G2D2 of 75 μL per vial (10 μM, OncoImmunin, Gaithersburg, MD, USA) at 37 °C for 30 min, followed by PBS washing twice. Meanwhile, the cleaved caspase substrate that was indicated for cell apoptosis showed the fluorescent emission peak at a wavelength of 580 nm by the excitation at a wavelength of 552 nm. Finally, cells were resuspended in 1 mL PBS per vial, and cell samples were collected and ready for measurement in a flow cytometry (BD FACSCanto II).

## 3. Results and Discussion

### 3.1. Characterization of the Flow Field

**Figure 3.**
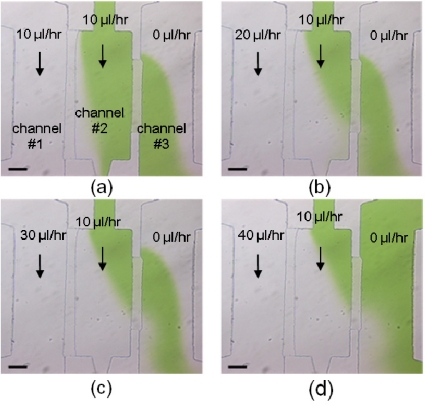
To test microfluidics for cell docking, the central flow rate is maintained at 10 μL/h, and the flow rates in a left channel were injected at: (**a**) 10 μL/h, (**b**) 20 μL/h, (**c**) 30 μL/h, (**d**) 40 μL/h. As the flow rate in a left channel increased, the central dye flow was visibly pushed to the right channel. Scale bar : 100 μm.

Figure 3 shows the dye flow test of the device for cell transportation and trapping in a reaction zone. The central dye flow for cell loading was introduced in a rate of 10 μL/h. In Figures 3(a)–(d), the flow rates in a left channel increased from 10 μL/h to 40 μL/h, while the right-channel flow was initially static. As the flow rate increased in a left channel, the central dye flow was pushed to the right channel by the pressure difference *via* the gap. It was also found by an increase of the flow rate up to 20 μL/h in a left channel that most of the central dye flow was pushed *via* the gap into the right channel. The flow test provided a reference of flow operation conducted for cell loading. As a result for cell trapping to be gentle, the flow field was designed for operation at 10 μL/h in a left channel and 10 μL/h for the central flow. With such an operation, microparticles and cells were trapped in the right side of the reaction zone. Moreover, to trap particles and cells at the left side of the reaction zone, the flows in the left channel were then turned off and the central flow rate was still maintained at 10 μL/h. With the pressure difference, the central flow in the central channel was found to pass through the gap to the left channel.

The pressure difference across the docked cells was derived from the calculation of flow rate across the gap, as shown in Table 1. As mentioned above, the pressure difference was directly proportional to the flow rate across the docked cells. Moreover, the shear force exerted on the cells was also calculated and listed in Table 1. According to the observation for cell docking, cells were deformed and pushed out through the gap structure when the flow rate in a left channel was greater than 10 μL/h. Meanwhile, the docked cells were easily squeezed through the gap when the force was greater than around two hundred piconewtons. The result in regard with the shear force is on the same order of magnitude as a reported study on force measurement of live cell deformation [27].

### 3.2. Particle and Cell Trapping by Microfluidic Manipulation

**Figure 4.**
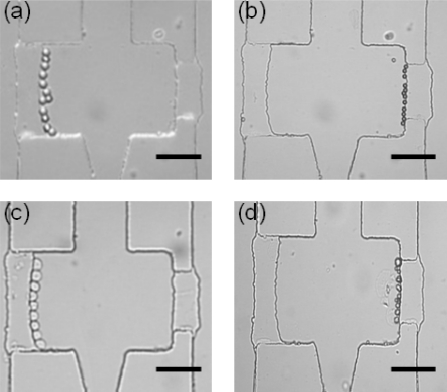
The photographs of particles in diameter of (**a**) 20 μm, and (**b**) 10 μm docked at the left and right sides, respectively. (**c**) The picture showed the cell docking of live K562 cancer cells. (**d**) The picture displayed the cell docking of live natural killer cells. Scale bar: 100 μm.

Two particle sizes of 10 μm and 20 μm in diameter were selected to simulate natural killer cells and cancer cells, respectively. Figure 4 shows the result of particle trapping conducted for simulation prior to actual cell loading. Particles of 20 μm in diameter began to be loaded with the central flow rate of 10 μL/h, while the flow rates were 15 μL/h and zero in the right and left channels, respectively. As shown in Figure 4(a), the particles were successfully trapped along the gap of the reaction zone due to the fluidic pressure drop between channels. After the particle trapping on one side of the reaction zone, the central flow still maintained its flow rate, but the flow rate of 15 μL/h in the right channel was turned off to be static. As a consequence of the pressure drop between channels, particles of 10 μm in diameter were loaded and then successfully docked at the right side of the reaction zone, as shown in Figure 4(b). Meanwhile, the number of particles trapped in the reaction zone depends upon the length of the gap.

The operation procedure has been verified with the cell device. Likewise, the cancer cells and natural killer cells were loaded and then docked at the left and right sides of the reaction zone, respectively. Figure 4(c) and (d) show the trapping of live cancer cells and natural killer cells. It was found that live natural killer cells docked at the gap tended to be aggregated naturally. In a longer gap structure, an aggregated group of live natural killer cells blocked the gap in a reaction zone. This may lead to problems in counting the NK cell number and mixing with cancer cells. As mentioned above, the docked cells were easily squeezed through the gap by a shear force greater than around 200 pN. Appropriate microfluidic operation is required in cell manipulation.

**Figure 5.**
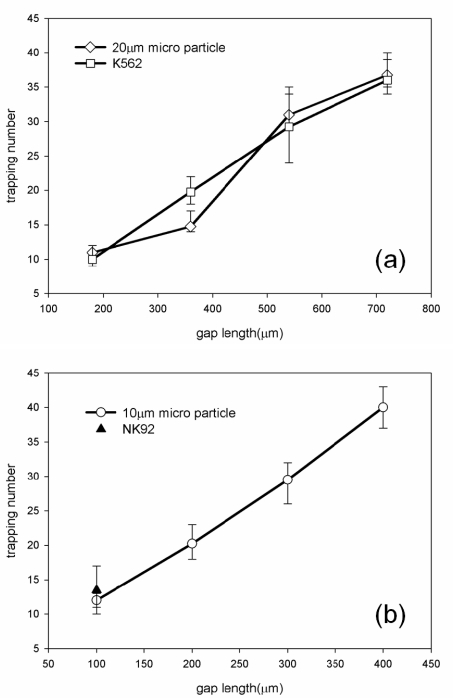
The relationship between the trapping number and the gap length. (**a**)The trapping numbers of 20-μm particles and K562 cells increased at the gap length from 200 μm to 700 μm. (**b**) Trapping number of 10-μm particles increased as the gap length increased.

Figure 5 shows the number of trapped particles and live cells associated with the gap length of the microdevice to determine cell quantities and cell ratio involved in cell-cell biological interaction. Meanwhile, both gaps of the reaction zone are designed with 1 μm in height to allow simple flow passage, but to trap particles and cells along the gap length. The gap lengths for 20-μm cells in a reaction zone were designed with 180 μm, 360 μm, 540 μm, and 720 μm, respectively. The cell ratio of effector (natural killer cell) to target (cancer cell) was designed to be 1 or 2. The gap lengths of 10-μm cells were designed with 100 μm, 200 μm, 300 μm, and 400 μm. In Figure 5(a), the result shows that the numbers of trapped particles and cancer cells were linearly proportional to the gap lengths. Those tests were conducted in three runs for experimental reproducibility. Moreover, Figure 5(b) shows that the numbers of 10-μm trapped particles and natural killer cells increased with the gap lengths of the reaction zones.

The result showed that micro particles of polystyrene successfully simulated the number of cell trapping for the effector of natural kill cells and the target of cancer cells in various gap lengths. This device has verified that various sizes and numbers of trapped cells can be determined by the gap lengths in a reaction zone of the cell device. It was found in a gap length of 100 μm that the culturing medium, growth environment, and impurity of natural killer cells tended to give a cluster aggregation form. Such an aggregation caused difficulties to the investigation at greater gap lengths. Culture medium and the environment need to be improved. Also, new sets of cultured natural killer cells are required.

### 3.3. Cell Characteristics and Cell-Cell Biological Interaction

**Figure 6.**
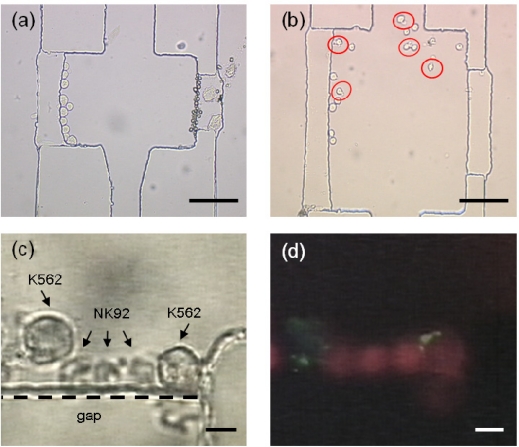
(**a**) The photograph showed both NK 92 and K562 cells docked in the reaction zone. (**b**) Cell-cell contact and interaction. Scale bar: 100 μm. (**c**) The close-up of conjugated natural killer and cancer cells, and (**d**) PhiPhiLux-G_2_D_2_ staining of cell apoptosis in cell-cell biological interaction after 2 hours. Scale bar: 10 μm.

Figure 6 shows the trapping of both heterogeneous cells and cell-cell contact by microfluidic manipulation in a reaction zone. Investigation of cell-cell biological interaction can be seen in movies SM1-SM3 in the supplementary information. It was found that there were 10 cancer cells and 17 natural killer cells trapped on both sides of the reaction zone in Figure 6(a).

As mentioned above, in the procedure for suspension blood cell in the manipulation microdevice, the air valves were pressed down by pneumatic pressures in the central channel to allow for a nearly closed micro environment in a reaction zone. Subsequently, the microfluidic manipulation was performed by pumping the culture medium into both left and right channels. Such fluidics resulted in cell departure from both gaps and thus cell motion inside the reaction zone. The cell motion increased the chance of cell-cell contact for heterogeneous suspension cells. With the cell characteristics of natural killer and cancer cells in leukemia, no multiple collisions or contacts were found, but cell-cell adhesion occurred upon cell-cell contact. Figure 6(b) shows the result of cell-cell adhesion and interaction of the cell device. It was found that one or multiple natural killer cells were conjugated with one large cancer cell. Cytotoxicity of NK cells ocurrs mainly through the granule exocytosis pathway where the perforin and granzyme B contents of granules are released into the immunological synapse after conjugate formation with the targets. In such a mechanism, the killing of cancer cells induces apoptosis by activation of the caspase cascade. In order to accurately identify the cancer cell death caused by NK cells, a specific staining of cell apoptosis due to NK-mediated killing is required. Figures 6(c) and (d) show magnified pictures of cell-cell interaction and cytolytic staining, respectively. As cancer cells were killed by NK cells, a specific NK-mediated killing biomarker of caspase-3 was present and could be stained for identification of cell apoptosis. The cell-cell interaction required approximate 2 hours. In this study, a specific caspase-3 biomarker used showed red fluorescence by PhiPhiLux-G_2_D_2_ staining in cell-cell biological interaction after 2 hours. Meanwhile, cancer cells labeled with QDs exhibited green fluorescence in monitoring. Moreover, the experiment was carried out to ensure the apoptosis of K562 executed by NK92 cells instead of the spontaneous cell death. The result showed that only two percent of K562 cells expressed red fluorescence by PhiPhiLux-G_2_D_2_ staining.

### 3.4. Cytotoxicity of NK Cells against Cancer Cells

Cytotoxicity of NK cells against cancer cells is the percentage of K562 cell lysis performed by NK cells to total K562 cells. In this study, the ratio of effector NK 92 cells to target K562 cells was set at 2 [2,26]. Meanwhile, the numbers of K562 cells docked in this microdevice were around 5 and 20 in gap lengths of 100 μm and 400 μm, respectively. Cell lysis of K562 cells caused by NK cells was identified through a specific caspase-3 biomarker in the presence of a cell apoptosis pathway. The biomarker can be stained by PhiPhiLux-G_2_D_2_ in cell-cell biological interaction after 2 hours.

**Figure 7.**
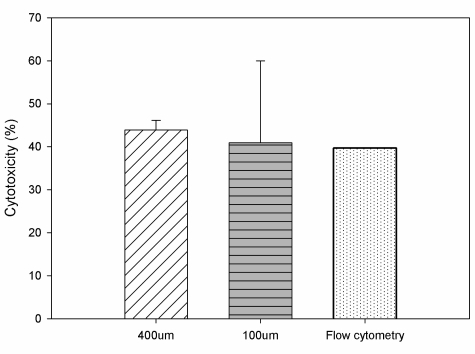
Cytotoxicity of effector NK92 cells against K562 target cells was around 40%. The experiments were performed with dilute cells (∼10^0^–10^2^ cells) in the microdevice with the 100-μm and 400-μm gap lengths, and a large amount of cells (∼10^6^ cells) in conventional flow cytometry analysis. The x-axis of the chart denotes micro device #1 in gap length of 400 μm (10^1^–10^2^ total trapped cells in cytotoxicity analysis), micro device #2 in gap length of 100 μm (10^0^–10^1^ total trapped cells in cytotoxicity analysis), and the flow cytometry of the conventional assay (∼10^6^ total cells in cytotoxicity analysis).

Figure 7 shows the cytotoxicity of NK cells against cancer cells for the events in cell manipulation microdevices and in the conventional approach. The cell manipulation microdevices required dilute cells (∼10^0^–10^2^ cells), while the conventional approach required around 1 × 10^6^ cells to be cultured in the dish and then measured in a flow cytometry analysis assay. The cytotoxicity of around 40% was found to be similar in the cases of dilute cells and a large cell population cells. Unlike the conventional approach, the device required the trapped cancer cells to be around 5 and 20 cells for experiment. It was also found for the gap length of 100 μm in the microdevice the result presented a larger variation of cytotoxicity due to the few cells involved. The result demonstrated a stable cytotoxicity for a gap length of 400 μm in the microdevice. With the cytotoxicity of NK cells against cancer cells, the cell manipulation microdevice has been preliminarily validated for such a specific effector to target ratio.

## 4. Conclusions

A new cell manipulation microdevice has been developed to allow heterogeneous suspensions of cells for cell docking, cell movement and cell-cell contact by microfluidic manipulation. The cell-based microdevice demonstrated the capability of monitoring cell interaction and apoptosis staining for examining the cytotoxicity of NK cells towards cancer cells. In cell loading, a flow rate of 10 μL/h was chosen for injection at the central and side flows such that both suspension cells could be gently docked at the gap structure in a reaction zone. The number of docking particles and cells was linearly proportional to the gap length. It was found that the trapping number can be controlled with gap length in this study. The experimental results demonstrated the presence of killed cancer cells by the PhiPhiLux staining technique. Finally, the cytotoxicity of around 40% was found to be similar in cases of dilute cells and a large population of cells. As a result, the cell manipulation microdevice has been preliminarily validated for a specific effector to target ratio, and exhibited its feasibility and potential for measuring the cytotoxicity of dilute cell suspensions.

## Supplementary Information



## Figures and Tables

**Table 1.** Calculations of the pressure difference and shear force across docked cells at the gap.

**Left inlet flow rate (μL/h)**	**Flow rate across the gap (μL/h)**	**Pressure difference (Pa)**	**Shear force (pN)**
10	3.9	0.07	101
20	11	0.21	288
30	21	0.42	556
40	31	0.63	813

The central flow rate is 10 μL/h.
