# Supplementary material for: Cell Docking, Movement and Cell-Cell Interactions of Heterogeneous Cell Suspensions in a Cell Manipulation Microdevice
Source: Sensors (Basel). 2011 Oct 12;11(10):9613–27. doi: 10.3390/s111009613 (PMC3231279; doi:10.3390/s111009613)
Supplement: Supplementary file 1 [file sensors-11-09613-s001.zip › sensors-11-09613-supplementary information.pdf]

Cell Docking, Movement and Cell-Cell Interactions of Heterogeneous Cell Suspensions in a Cell Manipulation Microdevice

Fei-Lung Lai [1], Yu-Hung Wang [1], Yu-Wei Chung [1], Shiaw-Min Hwang [2] and Long-Sun Huang [1,*]

[1] Institute of Applied Mechanics, National Taiwan University, 1 Sec. 4 Roosevelt Road, Taipei 10617, Taiwan; E-Mails: f95543034@ntu.edu.tw (F.-L.L.); yhwangx@ mems.iam.ntu.edu.tw (Y.-H.W.); ywchung@ mems.iam.ntu.edu.tw (Y.-W.C.)

[2] Bioresources Collection and Research Center, Food Industry Research and Development Institute, 331 Shih-Pin Road, Hsinchu 300, Taiwan; E-Mail: hsm@firdi.org.tw

* Author to whom correspondence should be addressed; E-Mail: lshuang@mems.iam.ntu.edu.tw; Tel.: +886-2-3366-5653; Fax: +886-2-2363-9290.

Received: 2 September 2011; in revised form: 22 September 2011 / Accepted: 29 September 2011 / Published: 12 October 2011

1. Cell Culture in the Micro Device

Figure S1. A series of images showing the culture of the K562 cells at fourth day after seeding. (**a**–**e**) The images of cell growth during culture from seeding to 4 days. In the experiment, none of K562 cells was determined by trypan blue staining.

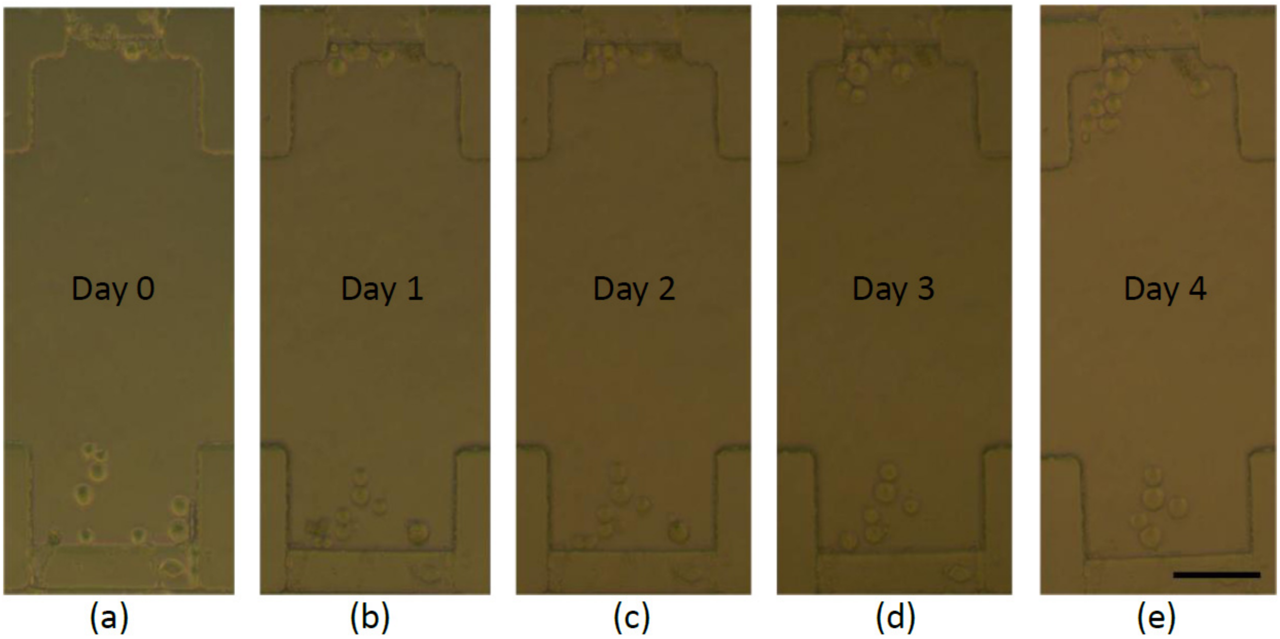

2. Air Valves Pressurization

Figure S2. Air valves were pressed down by pneumatic pressures in central channel to form a micro environment as reaction zone. The air valves bonding on the micro channel are located in back (**a**) and front (**b**) of reaction zone. (**c**–**d**) While the air valves are pressurized, the PDMS membranes are pressed down to block the channel.

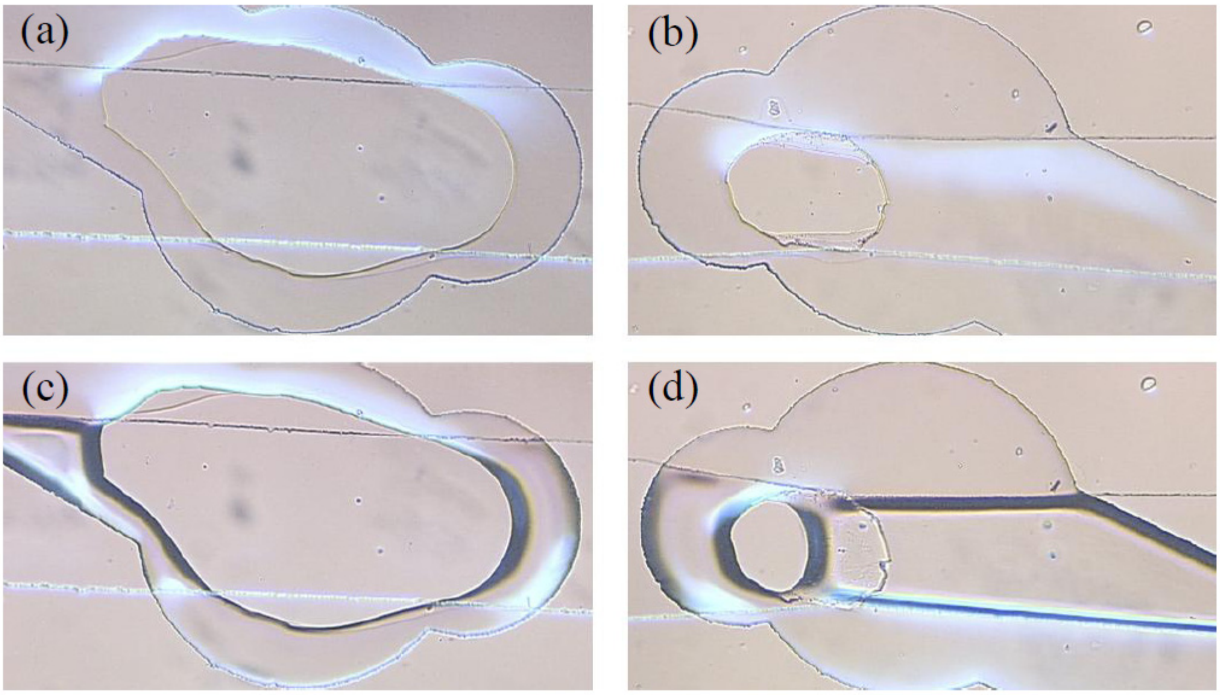

3. Computation of Dynamic Pressure, Major Loss and Minor Loss

Table S1. The calculation of dynamic pressure, major loss and minor loss for flow through contraction channel.

Left inlet flow rate (μL/h)	**Dynamic pressure** $\frac{V_{in}^2}{2} - \frac{V_{out}^2}{2}$ (10^{-6} m^2/s^2)	**Major loss** h_l (10^{-6} m^2/s^2)	**Minor loss** h_{lm} (10^{-6} m^2/s^2)
10	0.4	73.9	0.2
20	3.2	209.8	1.6
30	12.0	405.0	6.1
40	25.6	591.7	12.9

Supporting Information Movies

Supplementary movie 1:
Cell loading movie of target cells K562 docked beside bottom gap.
Supplementary movie 2:
Cell loading movie of effector cells NK92 docked beside top gap.

Supplementary movie 3:
Cell movement in the micro reaction zone by microfluidic manipulation.

© 2011 by the authors; licensee MDPI, Basel, Switzerland. This article is an open access article distributed under the terms and conditions of the Creative Commons Attribution license (http://creativecommons.org/licenses/by/3.0/).
